# Using a digital health tool to strengthen community engagement in arboviral disease prevention: an implementation study in Colombia

**DOI:** 10.1186/s12889-026-28576-7

**Published:** 2026-08-04

**Authors:** Maria Angelica Carrillo, Rocio Cardenas Sanchez, Susana Koo Chong, Tatiana Rivera Ramirez, Johanna Yañez, Winfried V. Kern, Jörg Lindenmeier, Axel Kroeger

**Affiliations:** 1 Centre for Planetary Health, Faculty of Medicine, University of Freiburg, Freiburg, Germany; 2 Research Group GIGA, Francisco de Paula Santander University, Cucuta, Colombia; 3 Vector Control Group, Departmental Health Institute of Norte de Santander-IDS, Cucuta, Colombia; 4 Division of Infectious Diseases, Department of Medicine II, Faculty of Medicine, University Medical Centre Freiburg, Freiburg, Germany; 5 Public and Non-Profit Management, Corporate Governance and Ethics, University of Freiburg, Freiburg im Breisgau, Germany

**Keywords:** Dengue, Chikungunya, Zika, *Aedes aegypti*, Digital health, WhatsApp, Disease prevention, Community engagement, Behaviour change, Coating, Vector control, Colombia

## Abstract

**Background:**

Arboviral diseases remain major public health problems in Colombia. Mobile applications such as WhatsApp are increasingly being used to support health interventions by improving community engagement and preventive practices. Given its widespread use in Colombia, this study aimed to evaluate a community-based public health strategy supported by WhatsApp to promote preventive practices and enhance community engagement in vector control activities, particularly the application of insecticidal coatings to water tanks.

**Methods:**

A quasi-experimental study with three groups (control, community visits only, and community visits plus WhatsApp) was conducted in two municipalities in Colombia. Around 300 households per group were recruited. Preventive practices and entomological indices were evaluated before and after the intervention using household surveys and larval/pupal inspections. Within-group changes were analysed using McNemar’s and Wilcoxon signed-rank tests, and intervention effects over time were estimated using difference-in-differences analysis. WhatsApp acceptability was also assessed using Likert-scale items.

**Results:**

A total of 874 households were included in the final analysis. The community visits and WhatsApp group showed significant improvements in preventive practices after the intervention, including proper management of small breeding sites (+ 15.9% points, *p* < 0.001). Reductions in vector abundance were observed in both intervention groups, but they were greater in the group that integrated WhatsApp; the House Index decreased by 22.8% points in the community visits plus WhatsApp group vs. 14.7% points in the community visits-only group. At 4 months, entomological indices were significantly lower in both intervention groups than in the control group, and no larvae or pupae were found in treated tanks. Community engagement related to coating application was high in the WhatsApp group, and WhatsApp was well accepted by community users, with most mean scores above 4.8 out of 5.

**Conclusions:**

The strategy combining community visits and WhatsApp within vector control activities was acceptable and more effective than community visits alone in improving preventive practices and reducing vector densities. The incorporation of WhatsApp may strengthen community engagement in prevention programmes in endemic urban settings.

**Trial registration:**

ClinicalTrials.gov, NCT07517731. Retrospectively registered on 8 April 2026.

**Supplementary Information:**

The online version contains supplementary material available at 10.1186/s12889-026-28576-7.

## Introduction

Arboviral diseases transmitted mainly by *Aedes* mosquitoes are an increasing global health threat, with over 5.6 billion people at risk worldwide [1]. Dengue, chikungunya, and Zika are among the most widespread arboviral diseases in urban settings and are transmitted mainly by the primary vector, *Aedes aegypti* [2, 3]. These diseases are characterized by recurrent outbreaks, severe health complications [4–7], and chronic sequelae associated with chikungunya [8].

In Colombia, there is co-circulation of four dengue virus serotypes and multiple lineages that have contributed to large outbreaks with higher fatality rates than the average reported in the Americas [9]. In 2019, more than 127,000 dengue cases were reported, exceeding the epidemic threshold in the country, with severe cases concentrated in the departments of Meta, Tolima, Huila, Santander, Cesar, Valle del Cauca, Sucre, Norte de Santander, Antioquia, and Casanare [10]. The chikungunya epidemic of 2014–2015 accounted for more than 450,000 reported cases and affected 96% of municipalities nationwide [11, 12]. Likewise, the Zika epidemic of 2015–2016 resulted in more than 90,000 suspected cases and numerous cases of congenital Zika syndrome, Guillain–Barré syndrome, and other neurological manifestations [13]. Norte de Santander is one of the states most affected by dengue, with more than 25% of households found to be positive for *Aedes* larvae or pupae in three municipalities: Cucuta, Villa del Rosario, and Los Patios [14].

The coexistence of dengue, chikungunya, and Zika, along with factors such as a hot climate, increasing population density, sanitation inequalities, deficiencies in vector control policies, poor household infrastructure, and human behaviours related to domestic water storage, increases the complexity of prevention efforts [15–19]. To combat these diseases, preventive strategies have relied on vertically managed programmes based on chemical, biological, and environmental vector control [20], as well as vaccine development [21]. However, these approaches face important limitations, including low levels of community participation, increasing insecticide resistance [22, 23], and the restricted effectiveness of the currently available vaccine [24, 25]. These challenges highlight the need to shift toward more effective approaches that actively integrate communities into sustained source reduction efforts.

To address these challenges, an innovative vector control tool, known as insecticidal coating (IC), was applied to laundry tanks in Norte de Santander, Colombia, to disrupt larval and pupal development and cause mortality in adult mosquitoes [26]. This dual effect results from the combined action of two microencapsulated active ingredients, alphacypermethrin and pyriproxyfen, which allow their gradual release in water containers. A recent study conducted in Cucuta, Colombia, showed its efficacy for up to 12 months in inhibiting the emergence of larvae and pupae [26]. However, its sustained effectiveness depends heavily on community engagement, since residents must empty the tanks and keep them dry for approximately one day to ensure proper adhesion of the product to the inner walls.

In recent years, digital interventions using mobile phones have emerged as promising tools to strengthen community engagement in the prevention and control of dengue, chikungunya, and Zika [27]. Recent experiences in Asia show that mobile surveillance and communication platforms can mobilize communities and generate innovative local solutions for dengue prevention, even in resource-limited settings [27, 28]. In the Americas, mobile platforms such as DengueChat and ZIKAMOB have shown encouraging results in reinforcing preventive behaviours through reminders, education, and digital support for students and community members [29, 30]. More recently, a feasibility study in Colombia showed that sending WhatsApp messages to women in urban communities was acceptable and usable. In addition, the app improved intentions to adopt key preventive practices, such as weekly cleaning of water tanks [31].

Despite this increasing evidence, no structured public health strategy explicitly grounded in behaviour change principles and integrating both a digital component and local vector control strategies, such as IC, has yet been evaluated at the community level. In a context where more than 90% of households own a smartphone and use WhatsApp daily [32, 33], this application may represent a valuable opportunity to strengthen health behaviours and community engagement in the control of dengue and other arboviral diseases. Therefore, to address this gap, the present study incorporated WhatsApp as an additional health promotion and behavioural reinforcement component within a community-based public health strategy designed to support IC application in laundry tanks and to foster community engagement in arboviral disease prevention.

## Methods

### Aim

The purpose of this study was to assess changes in preventive practices, vector abundance, and community engagement in vector control activities following a community-based public health strategy supported by WhatsApp in two municipalities in Colombia.

### Study setting

The study was conducted in the Metropolitan Area of Cucuta, in the department of Norte de Santander. This region is located in the valley of the same name, at the foot of the Eastern Cordillera of the Colombian Andes. The Metropolitan Area is composed of the municipalities of Villa del Rosario, Los Patios, El Zulia, San Cayetano, and Puerto Santander, and has temperatures ranging from 28 to 37 °C [34]. For this study, the municipalities of Los Patios and Villa del Rosario were selected because they have been historically affected by dengue, chikungunya, and Zika, with high vector densities and documented resistance in *Aedes* mosquitoes [14, 35]. According to projections by the National Administrative Department of Statistics (DANE), these two municipalities have a combined projected population of 232,053 inhabitants in 2026. Villa del Rosario represents 7.3% of the total population of Norte de Santander, while Los Patios accounts for 6.1% [36].

### Study design

This study was an implementation study that used a quasi-experimental design with three groups: one control group, one community visits-only (CV only) group, and one community visits plus WhatsApp (CV + WA) group. Both intervention groups received the insecticidal coating. The three groups were geographically separated to reduce the risk of contamination. The study measured changes in respondents’ preventive practices and vector abundance at the household level through pre- and post-intervention surveys. In addition, community engagement was assessed through observations recorded by the field team, and acceptability was evaluated using additional questions in the post-intervention survey.

The study was conducted over a one-year period, from September 2021 to September 2022. The pre-intervention survey was completed in September 2021, WhatsApp message delivery started in mid-October 2021, and IC was applied in December 2021. A monitoring assessment was conducted 4 months after IC application, and the post-intervention survey was completed in September 2022.

### Study participants

**Fig. 1:**
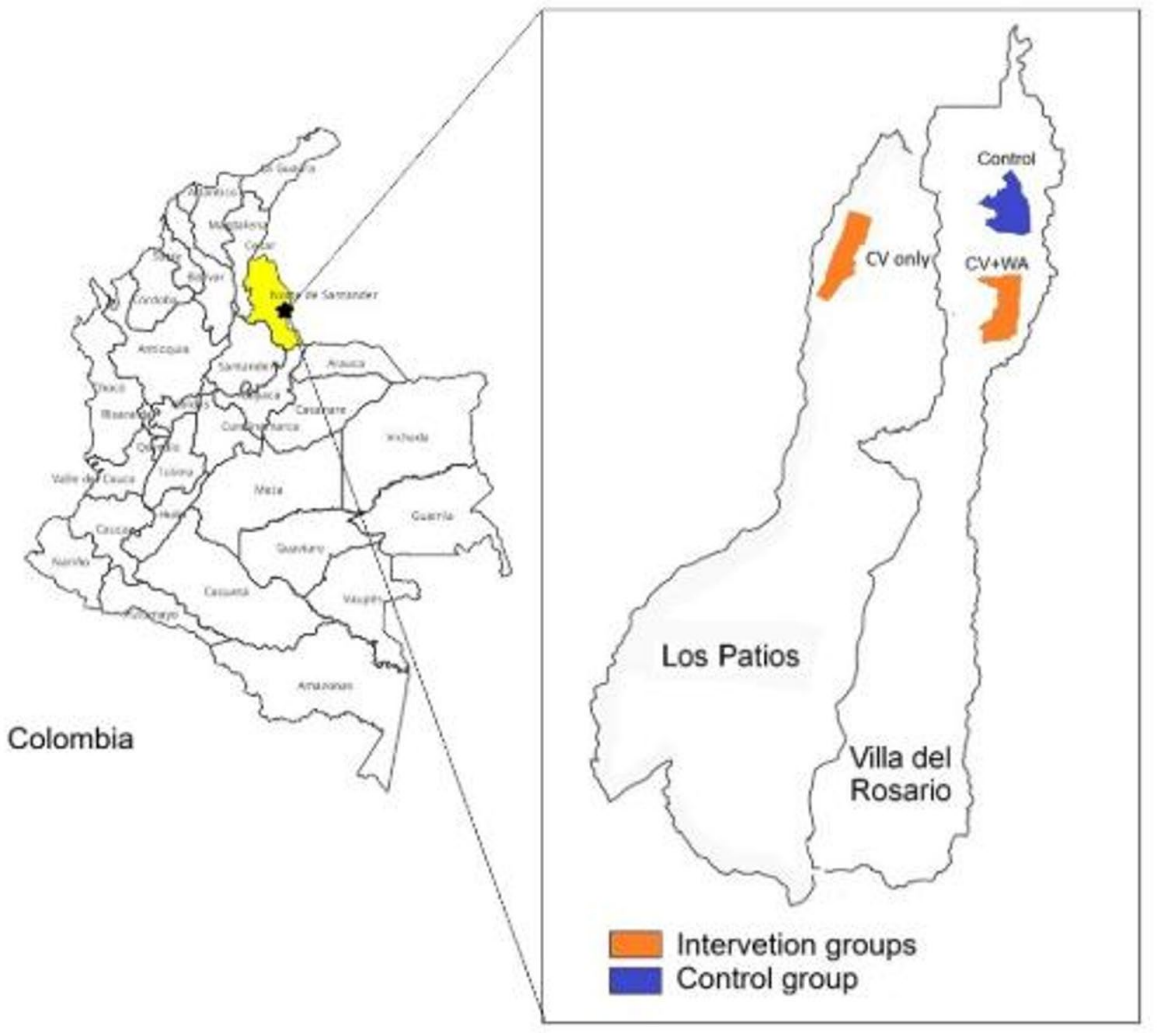
Map of the study area showing intervention and control groups in Los Patios and Villa del Rosario. CV only: community visits-only group; CV + WA: community visits plus WhatsApp group. This figure was created using QGIS software

With support from the regional Ministry of Health (MoH), highly endemic neighbourhoods were identified and mapped. These neighbourhoods had similar sociodemographic characteristics and comprised approximately 200 to 400 households each. They were grouped into three large study groups located in different corners of the study area; in particular, the two intervention groups were separated, with one located in each municipality (Fig. 1). A lottery method was used to assign the study groups. Each study group consisted of approximately four to five neighbourhoods. From each household, one adult member was recruited as a study participant and survey respondent. Eligible respondents had to own a smartphone with WhatsApp installed, be able to receive and read messages, reside within the study group, and have a laundry tank in their household. Respondents also reported sociodemographic information for all household members.

#### Sample size and sampling technique

**Fig. 2:**
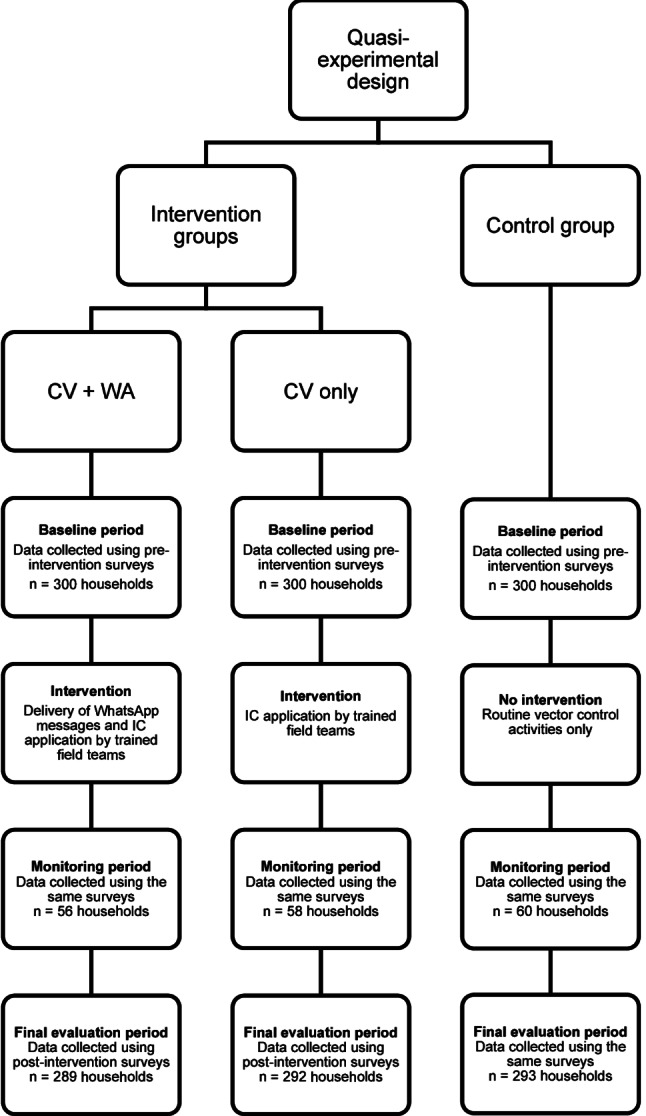
Flow diagram of the study design. CV only: community visits-only group; CV + WA: community visits plus WhatsApp group

The study recruited 300 households per group using a sampling interval of 8 households, with one adult member selected from each household. This sample size was considered adequate to achieve a statistical power of 95% and to detect a difference of at least 15% in preventive practices between groups. The sample size was calculated using a proportions-based approach with OpenEpi software (version 3.01) [37]. Therefore, a total of 900 households were initially enrolled in the study (Fig. 2). All selected households in the three study groups were visited for the pre-intervention survey. An operational random subsample of approximately 60 households per group was visited by the field team to monitor the intervention after 4 months, resulting in 174 households in total (56 in the CV + WA group, 58 in the CV-only group, and 60 in the control group). This number represented approximately 20% of the group sample and was considered feasible for field monitoring given the logistical constraints and available time. This subsample was selected using Excel’s randomization function. At the final evaluation, all 900 households initially enrolled in the study were revisited. However, 26 households did not take part in the post-intervention survey; therefore, the final analysis of preventive practices and entomological outcomes included data from 874 households.

### Intervention components

The interventions in this study included: (a) health promotion through community visits (CV), during which a field team distributed stickers and flyers about the IC, and (b) community visits combined with WhatsApp reminders sent via smartphone (CV + WA).

Health promotion through community visits was carried out by field teams composed of community volunteers and health workers from each study group. These teams received one week of training from the principal investigator (PI) on how to announce the intervention, provide instructions on IC application, and distribute the promotional materials needed to support IC application in tanks. The materials were designed according to the product application procedure and based on previous application tests conducted by the Freiburg research team [26]. In addition, loudspeakers were used as part of the promotional activities in both intervention groups.

Three months after completion of the pre-intervention survey, IC application began and lasted approximately three to four weeks in each group. On the day before IC application, health promotion activities were carried out in each household in both the CV-only and CV + WA groups during regular home visits.

#### WhatsApp reminder and delivery

The WhatsApp reminder component consisted of health prevention and promotion messages based on arboviral disease prevention information developed by the Colombian campaigns [38] and on the technical specifications for the IC provided by the manufacturer (Inesfly Co., Spain). The present study used the same message content and functions that were developed, tested, and described in detail in a previous feasibility study [31]. These messages had four main functions: (i) to educate participants about general and relevant concepts related to arboviral diseases; (ii) to promote and encourage participants to use IC; (iii) to reinforce IC application and other preventive practices; and (iv) to maintain regular communication with participants through general project-related information [31].

A total of 45 community members validated and tested these messages during the feasibility study, while health experts reviewed and validated them through a workshop [31]. Ten additional messages were later included to provide specific information about application dates and further instructions regarding the coating. These messages are described in Supplementary File 1. The messages were short, with approximately 350 characters each. All messages were written in Spanish and were reviewed and approved by coordinators from MoH in Norte de Santander. Further details about the messages are provided in the previous research project [39].

Message delivery started six weeks after the pre-intervention survey, with approximately one message sent per week. However, on the day before IC application, two reminder messages were sent on the same day, one in the morning and one in the afternoon, to reinforce preparation for IC application. After IC application to tanks, participants in the CV + WA group continued receiving WhatsApp messages, with a total of 30 messages delivered during the 35-week intervention period. Messages were usually sent on Tuesdays, Wednesdays, and Thursdays, according to the schedule of each neighbourhood, and were generally delivered in the morning by a facilitator trained by the PI [39].

Participants did not incur any direct costs for receiving the messages, as WhatsApp is a free application that uses internet connectivity. Participants were encouraged to save the project phone number to ensure that they received the messages successfully.

Participants in the control group did not receive any intervention (neither IC nor WhatsApp). They only received the standard routine visits carried out by the vector control programme [39].

### Research instruments

This study employed three different research instruments for quantitative data collection. The pre- and post-intervention surveys consisted of a household questionnaire and a larval/pupal household survey. The household questionnaire was used and adapted from published studies in Colombia [14, 31, 40]. This questionnaire collected sociodemographic and health data, as well as household practices of participants. In addition, questions were added to assess the acceptability of messages, based on a previous study conducted in Nepal [41]. This household questionnaire is available in Supplementary File 2.

The larval/pupal household survey was developed by the Special Programme for Research and Training in Tropical Diseases (TDR) of the World Health Organization (WHO) [42]. This standard survey was adapted to include specific breeding sites relevant to the study setting. This instrument is available in Supplementary File 3. Both instruments (household questionnaire and larval/pupal household survey) were used in the pre-intervention, monitoring (after 4 months), and post-intervention periods (after 9 months). Five university students trained by the research team conducted the household questionnaire, while, in parallel, five health workers carried out the entomological inspections and recorded the breeding sites in the larval/pupal household survey.

The third research instrument was employed during the intervention, specifically during IC application in tanks. The research team developed a field form to record households that accepted IC application and followed the instructions (e.g. emptying and cleaning the tank) in the CV-only and CV + WA groups. This form is available in Supplementary File 4. All research instruments were pre-tested and modified based on feedback from members of the vector control programme in each municipality to check flow, consistency, and validity.

### Statistical analysis

This study used RStudio software (R Foundation, version 4.4.2) for data management and statistical analysis of quantitative data. Sociodemographic characteristics were summarized for all household members reported by the adult respondent, while preventive practices, community engagement indicators, and entomological outcomes were analysed at the household level. Categorical variables were summarized as frequencies and percentages with confidence intervals, whereas continuous variables were described using means and standard deviations (SD). Chi-square tests were used to compare baseline sociodemographic characteristics and other categorical variables (i.e. IC application, willingness to paint again, and tank emptying) between study groups.

For preventive practices, proportions were calculated before and after the intervention in each study group. McNemar’s test was used to assess within-group changes in paired categorical responses. In addition, difference-in-differences (DiD) analyses using linear probability models were conducted to estimate the effect of the intervention over time at the household level. Comparisons of binary practice variables across the three groups were performed using chi-square tests. Multivariable logistic regression models were also conducted to identify factors associated with preventive practices after the intervention, adjusting for potential confounders such as sex, age, and education.

Entomological indices (the House Index: HI, Container Index: CI, Breteau Index: BI, and Pupae per Person Index: PPI) were analysed by cluster to assess changes in vector abundance during the pre- and post-intervention periods. These indices were calculated following the WHO standard operating procedures [42] and the pupal count methodology described by Romero-Vivas [43]. Normality of the pre–post changes was assessed using the Shapiro–Wilk test. As the changes in CI, BI, and PPI were not normally distributed in any of the three groups (all *p* < 0.001), within-group comparisons were performed using the Wilcoxon signed-rank test. For HI, which was analysed as a paired binary outcome, McNemar’s test was employed. Changes across groups were further analysed using difference-in-differences analysis. Comparisons between groups were performed using chi-square tests for HI and CI, and Poisson-based methods for BI and PPI.

Acceptability of the WhatsApp intervention was assessed using 5-point Likert-scale items based on a previous study [41]. Each item (perceived usefulness, enjoyment, and satisfaction) was rated on a scale from 1 = strongly disagree to 5 = strongly agree. Mean scores and standard deviations were calculated for each acceptability item, and average agreement scores were used to summarize participants’ perceptions of the intervention. Higher scores (> 3) indicated agreement or positive acceptability. All *p*-values were considered statistically significant at *p* < 0.05.

## Results

### Sociodemographic characteristics of residents

Sociodemographic information on residents was obtained from an adult respondent in each household. Most respondents were women (71.2%). The mean household size was 4 residents (SD = 1.7). The field team collected data on 3,252 residents in this study (Table 1). Most residents were female (53.7%), and the largest age group was 35–64 years (36.5%). More than half had completed secondary or higher education (57.3%) and reported remaining within their neighbourhood during the day (67.8%).

**Table 1:** Sociodemographic characteristics of residents

Characteristics of residents		Total *n* (%)	Control *n* (%)	CV only *n* (%)	CV + WA *n* (%)	*p*-value
Total residents		3,252 (100)	1,062 (100)	1,051 (100)	1,139 (100)	
Sex	Female	1,745 (53.7)	564 (53.1)	576 (54.8)	605 (53.1)	0.664
	Male	1,507 (46.3)	498 (46.9)	475 (45.2)	534 (46.9)	
Age group in years	< 1	32 (1.0)	14 (1.3)	5 (0.5)	13 (1.1)	0.053
	1–4	146 (4.5)	58 (5.5)	44 (4.2)	44 (3.9)	
	5–9	251 (7.7)	93 (8.8)	65 (6.2)	93 (8.2)	
	10–19	484 (14.9)	146 (13.7)	150 (14.3)	188 (16.5)	
	20–34	719 (22.1)	238 (22.4)	232 (22.1)	249 (21.9)	
	35–64	1,187 (36.5)	376 (35.4)	396 (37.7)	415 (36.4)	
	> 64	433 (13.3)	137 (12.9)	159 (15.1)	137 (12.0)	
Education level	No formal education	124 (4.1)	36 (3.7)	45 (4.5)	43 (4.0)	0.002
	Primary	1,171 (38.6)	416 (43.0)	340 (34.0)	415 (38.9)	
	Secondary above	1,738 (57.3)	515 (53.3)	614 (61.5)	609 (57.1)	
Mobility status	Within-group^a^	2,202 (67.8)	707 (6.9)	726 (69.1)	769 (67.5)	0.519
	Outside-group^b^	1,044 (32.2)	350 (33.1)	324 (30.9)	370 (32.5)	

Sociodemographic characteristics are based on all household members reported by the adult respondent

^a^Within-group mobility = residents who stayed mostly within their neighbourhood during the day

^b^Outside-group mobility = residents who spent most of the day outside their neighbourhood during the day

### Monitoring after IC application

Four months after IC application in tanks, the field team visited a total of 174 households and collected data from 756 residents in the three groups (Table 2). Vector control workers inspected a total of 596 water containers (CV + WA = 188; CV only = 166; control group = 240). Entomological indices were significantly lower in the intervention groups, with the CV + WA group showing the lowest values (e.g. HI = 8.9%; *p* = 0.0005), while the control group consistently had the highest indices (e.g. HI = 33.3%). No larvae or pupae were observed in treated tanks in either intervention group. In addition, most users (76.8%) perceived WhatsApp as a useful digital tool for delivering health promotion information related to arboviral disease prevention (mean score: 4.7/5), and no technical problems were reported.

**Table 2:** Entomological indices in the three groups 4 months after IC application

Variable	Total	Control	CV only	CV + WA	*p*-value
Households inspected	174	60	58	56	-
Residents involved	756	288	217	251	-
HI [CI 95%]	17.8% [12.4%-24.3%]	33.3% [21.7%-46.7%]	10.3% [3.9%-21.2%]	8.9% [2.9%-19.6%]	0.0005^a^
CI [CI 95%]	8.3% [6.2%-10.8%]	14.2% [10.0%-19.2%]	6.0% [2.9%-10.8%]	2.7% [0.9%-6.1%]	< 0.001^a^
BI [CI 95%]	28.2 [21.6–35.5]	56.7 [43.2–69.4]	17.2 [8.59–29.4]	8.9 [3-19.6]	< 0.001^b^
PPI [CI 95%]	0.19 [0.16–0.22]	0.44 [0.38–0.50]	0.05 [0.02–0.09]	0.03 [0.01–0.06]	< 0.001^b^

Entomological indices were calculated and analysed at the household level

^a^HI and CI were compared across groups using chi-square tests

^b^BI and PPI were compared as rate-based indices using Poisson-based comparisons of aggregated counts

### Effects of the intervention on key preventive practices

**Fig. 3:**
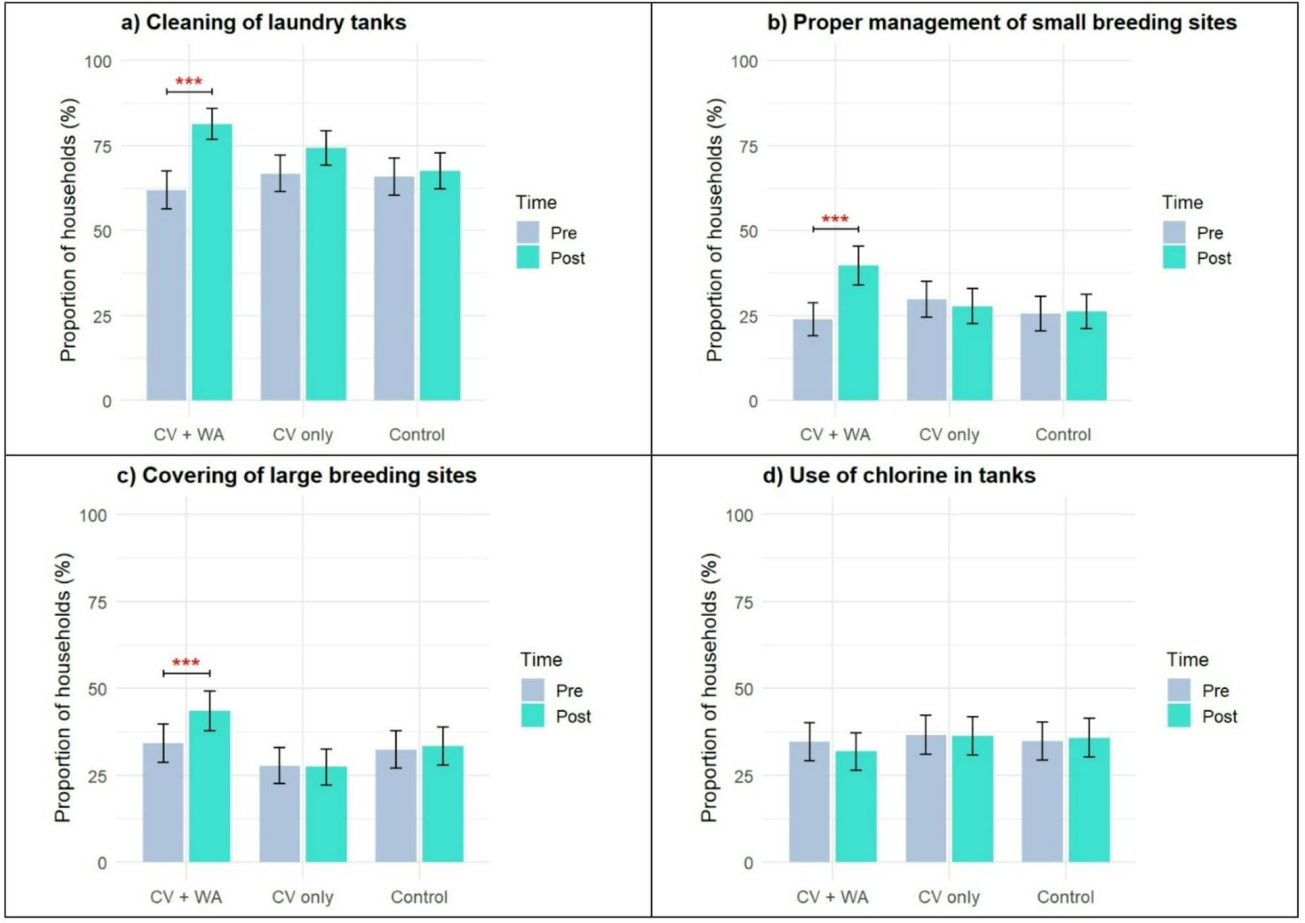
Effects on key practices in the three study groups. Bars show the proportion of households reporting each practice before and after the intervention. *** indicates *p* < 0.001. Graphs were created using RStudio software (version 4.4.2).

Pre- and post-intervention proportions of key preventive practices are presented in Table 3. Initially, there were no statistically significant differences in pre-intervention practices between the study groups. After the intervention, the highest increases in key preventive practices were observed only in the CV + WA group, with significant percentage-point increases of 19.4 in cleaning laundry tanks, 15.5 in the proper management of small breeding sites, and 9.3 in the covering of large breeding sites (*p* < 0.001). No change was observed in the use of chlorine in tanks in the three groups (Fig. 3).

DiD analysis showed significant improvements in the CV + WA group compared with the CV-only group and the control group combined for management of small breeding sites (DiD = 16.6% points), covering of large water breeding sites (DiD = 9.0), and cleaning laundry tanks (DiD = 14.8), but not for chlorine use.

**Table 3:** Preventive practices before and after the intervention in the three study groups

Practice	Estimates	Control	CV only	CV + WA	DiD^c^
Cleaning laundry tanks	Pre-intervention % [95% CI]	65.9% [60.4%, 71.3%]	66.8% [61.4%, 72.2%]	61.9% [56.3%, 67.5%]	
	Post-intervention % [95% CI]	67.6% [62.2%, 72.9%]	74.3% [69.3%, 79.3%]	81.3% [76.8%, 85.8%]	
	Difference	1.7^a^	7.5^a^	19.4^a^	**14.8**
	*p*-value	0.733^b^	0.066^b^	< 0.001^b^	**0.001**
Proper management of small breeding sites	Pre-intervention % [95% CI]	25.6% [20.6%, 30.6%]	29.8% [24.5%, 35%]	23.9% [19%, 28.8%]	
	Post-intervention % [95% CI]	26.3% [21.2%, 31.3%]	27.7% [22.6%, 32.9%]	39.4% [33.8%, 45.1%]	
	Difference	0.7^**a**^	-2.1^**a**^	15.9^**a**^	**16.6**
	*p*-value	0.752^b^	0.114^b^	< 0.001^b^	**< 0.001**
Covering of large breeding sites	Pre-intervention % [95% CI]	32.4% [27.1%, 37.8%]	27.7% [22.6%, 32.9%]	34.3% [28.8%, 39.7%]	
	Post-intervention % [95% CI]	33.4% [28%, 38.8%]	27.4% [22.3%, 32.5%]	43.6% [36.9%, 48.3%]	
	Difference	1.0^**a**^	-0.3^**a**^	< 0.001^**a**^	**9.0**
	*p*-value	0.863**	1	0.016^b^	**< 0.001**
Use of chlorine in tanks	Pre-intervention % [95% CI]	34.6% [29.1%, 40.1%]	36.6% [31.1%-42.2%]	34.8% [29.4%, 40.3%]	
	Post-intervention % [95% CI]	31.8% [26.5%, 37.2%]	36.3% [30.8%, 41.8%]	35.8% [30.3%, 41.3%]	
	Difference	-2.8^**a**^	-0.3^**a**^	1.0^**a**^	**-3.1**
	*p*-value	0.523^b^	1^b^	0.863^b^	**0.514**

Preventive practices were assessed at the household level, based on responses from one adult respondent per household

^a^Percentage-point difference between post-intervention and pre-intervention values within each group

^b^*p*-values for within-group pre–post comparisons were obtained using McNemar’s test

^c^DiD estimates compare the CV + WA group with the CV-only group and the control group combined. Estimates in the column DiD were obtained from linear probability models with robust standard errors clustered at the household level

In addition, multivariable logistic regression showed that the CV + WA group had higher odds of adopting most preventive practices than the control group. In contrast, chlorine tablet use was not associated with the intervention and showed only limited associations with selected sociodemographic characteristics [Supplementary File 5].

### Effects of the intervention on vector abundance

**Fig. 4:**
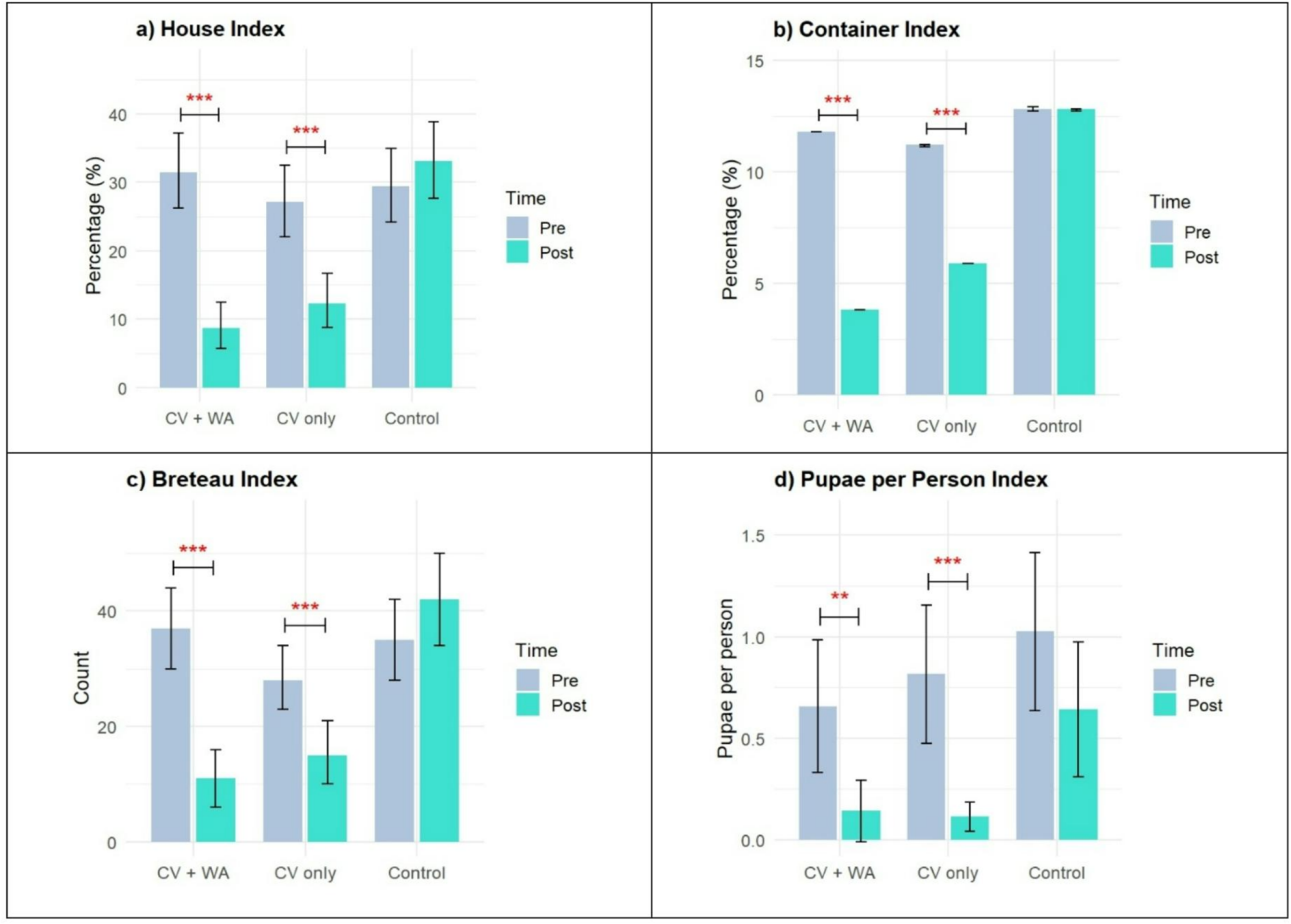
Effects of the intervention on vector abundance in the three study groups. Bars represent mean values of entomological indices before and after the intervention, with 95% confidence intervals. *** indicates *p* ≤ 0.001 and ** indicates *p* ≤ 0.01. Graphs were created using RStudio software (version 4.4.2)

Pre- and post-intervention entomological proportions are presented in Table 4. Before the intervention, high vector abundance was found in the three groups. After the intervention, the highest reductions in entomological indices were observed in the CV + WA group, followed by the CV-only group. HI decreased by 22.8% points in the CV + WA group and by 14.7% points in the CV-only group, while it increased by 3.8% points in the control group. Similar reductions were observed for other indices in the intervention groups (Fig. 4). No improvements were observed in the control group. In addition, DiD analysis showed significant reductions in the CV + WA group compared with the CV-only group and the control group combined for HI (DiD = -17.4), CI (DiD = -5.3), and BI (DiD = -0.23).

**Table 4:** Entomological indicators before and after the intervention in the three study groups

Indicator	Estimates	Control	CV only	CV + WA	DiD^c^
**House Index**	Pre-intervention % [95% CI]	29.4% [24.2, 34.9]	27.1% [22.0, 32.5]	31.5% [26.2, 37.2]	
	Post-intervention % [95% CI]	33.1% [27.7, 38.8]	12.3% [8.8, 16.7]	8.7% [5.7, 12.5]	
	Difference	3.8^a^	-14.7^a^	-22.8^a^	**-17.4**
	*p*-value	0.371^b^	< 0.001^b^	< 0.001^b^	**< 0.001**
**Container Index**	Pre-intervention % [95% CI]	12.8% [10.2, 15.4]	11.2% [8.8, 13.5]	11.8% [9.6, 14.0]	
	Post-intervention % [95% CI]	12.8% [10.2, 15.3]	5.9% [3.8, 8.0]	3.8% [2.2, 5.4]	
	Difference	-0.1^a^	-5.2^a^	-8.0^a^	**-5.3**
	*p*-value	0.911^b^	0.001^b^	< 0.001^b^	**0.004**
**Breteau Index**	Pre-intervention [95% CI]	0.35 [0.28, 0.42]	0.28 [0.23, 0.34]	0.37 [0.30, 0.44]	
	Post-intervention [95% CI]	0.42 [0.34, 0.50]	0.15 [0.10, 0.21]	0.11 [0.06, 0.16]	
	Difference	0.07^a^	-0.13^a^	-0.26^a^	**-0.23**
	*p*-value	0.176^b^	< 0.001^b^	< 0.001^b^	**< 0.001**
**Pupae per Person Index**	Pre-intervention [95% CI]	1.026 [0.636, 1.415]	0.817 [0.476, 1.157]	0.657 [0.331, 0.984]	
	Post-intervention [95% CI]	0.642 [0.310, 0.975]	0.113 [0.040, 0.186]	0.141 [-0.010, 0.293]	
	Difference	-0.384^a^	-0.704^a^	-0.516^a^	**0.027**
	*p*-value	0.115^b^	< 0.001^b^	< 0.001^b^	**0.911**

Entomological indices were calculated and analysed at the household level

^a^Percentage-point difference between post-intervention and pre-intervention values within each group. For HI and CI, differences are expressed in percentage points; for BI and PPI, differences are expressed in index units

^b^*p*-values for the Control, CV-only, and CV + WA groups were obtained using McNemar’s test for HI and Wilcoxon signed-rank tests for CI, BI, and PPI

^c^DiD estimates compare the CV + WA group with the CV-only group and the control group combined. Estimates in the column DiD were obtained from linear probability models with robust standard errors clustered at the household level

### Community engagement

**Fig. 5:**
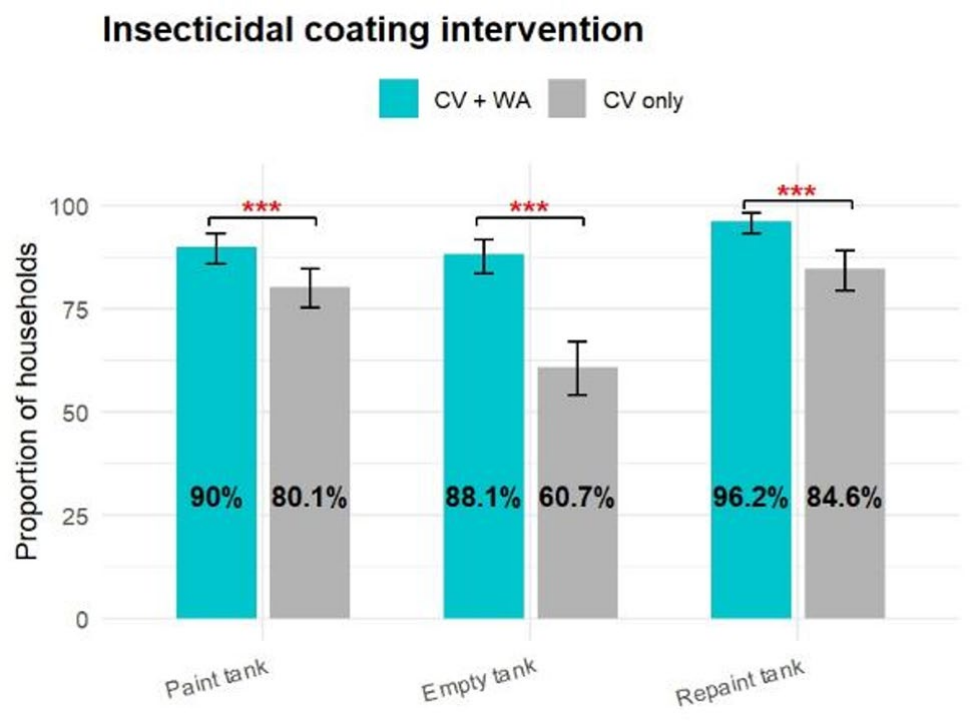
Community engagement during IC application. Bars represent the proportions of households that followed the instructions during household visits for the IC intervention, with 95% confidence intervals. *** indicates *p* ≤ 0.001. The graph was created using RStudio software (version 4.4.2)

Community engagement in vector control activities was particularly evident during IC application. During household visits in both groups, the field team observed and recorded households that followed the instructions, particularly emptying and cleaning the tanks and allowing IC to be applied. Willingness to reapply the product was also assessed. All three indicators were higher in the CV + WA group than in the CV-only group (Fig. 5).

### Perceptions of WhatsApp

**Fig. 6:**
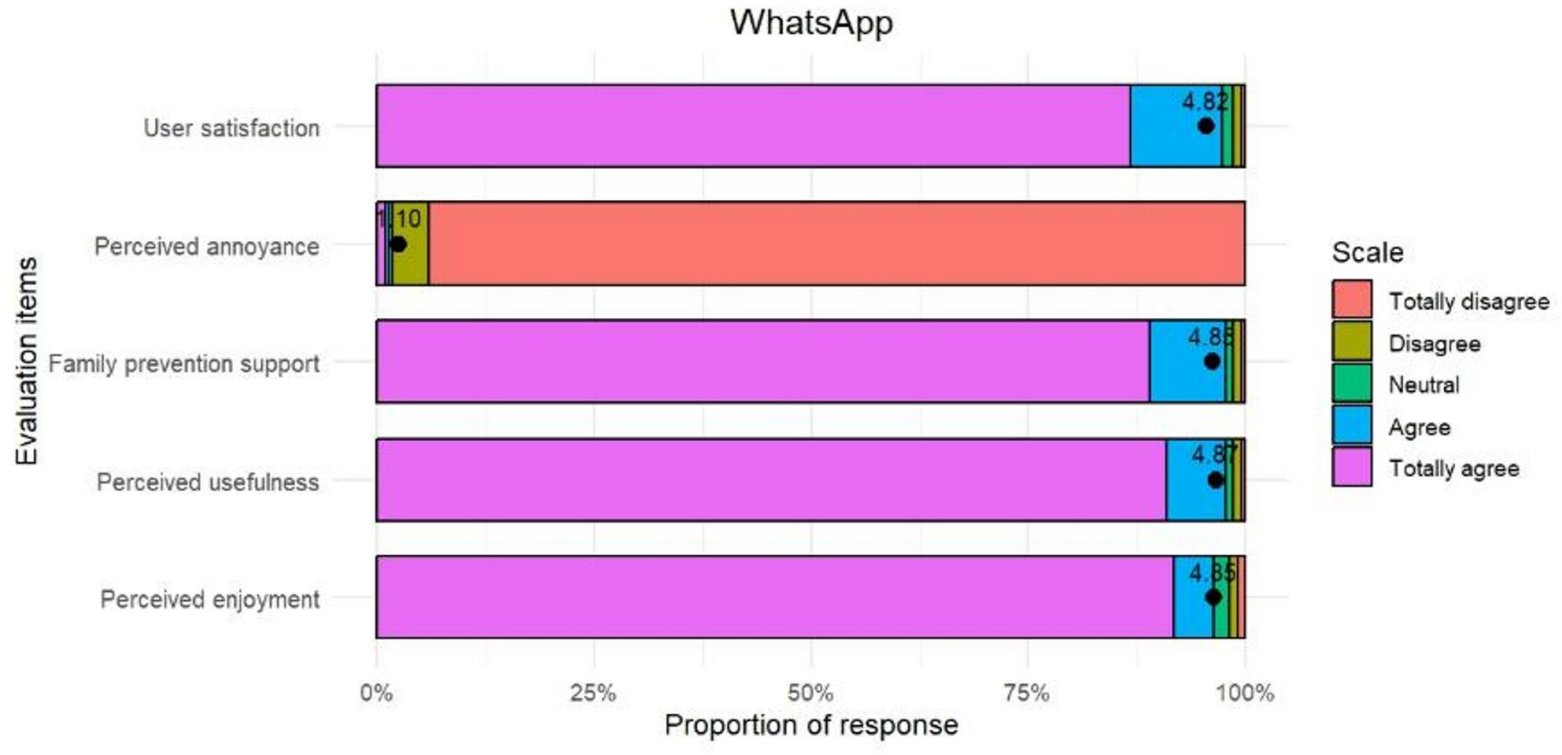
Responses to acceptability items using a 5-point Likert Scale. Bars show the distribution of responses across five items assessing acceptability on a 5-point Likert scale, ranging from totally disagree to totally agree. Black dots indicate the mean score for each item. The graph was created using RStudio software (version 4.4.2)

High ratings were observed for most items of WhatsApp acceptability, particularly perceived usefulness (4.87), family prevention support (4.80), perceived enjoyment (4.85), and user satisfaction (4.82) (Fig. 6). Responses to these items were predominantly positive, with most participants selecting “totally agree” or “agree.” In contrast, the item related to perceived annoyance received a very low mean score (1.10), indicating that participants generally did not find the messages annoying.

## Discussion

This implementation study evaluated a community-based public health strategy supported by WhatsApp to promote key practices for arboviral disease prevention and community engagement in vector control activities in endemic urban communities in Colombia. The CV + WA intervention group showed the greatest improvements in key preventive practices. Larval and pupal indices decreased in both intervention groups compared with the control group, but the reduction was significantly greater in the group that integrated WhatsApp (CV + WA group). Strong community engagement was reflected in the high proportion of households that participated in IC application and followed the instructions delivered through WhatsApp and community visits. In addition, community users gave positive ratings to the WhatsApp messages, suggesting that this app was well accepted in urban communities in Colombia.

Digital technologies have been increasingly used to support health promotion, including in low-resource settings [44]. For arboviral disease prevention, SMS-based interventions have shown potential to improve household practices and, in some settings, reduce *Aedes* indices in Nepal and Peru [41, 45]. However, unidirectional SMS-only interventions may limit interactivity, which can make sustained engagement more challenging, particularly in remote settings [46]. As smartphone access has expanded, researchers have developed digital systems, including web-based platforms and mobile apps for dengue prevention and control (e.g., VECTOS, VazaDengue, MEWAR) [47–49]. In this study, rather than developing a new application, we leveraged a familiar and widely used platform. WhatsApp, Facebook Messenger, and WeChat enable rapid two-way communication and multimedia sharing, which can strengthen message reinforcement and user engagement [50]. Given the need to reach many households and engage the community in the vector control operations, WhatsApp was chosen as a two-way communication tool in areas where smartphones and WhatsApp were widely used. This approach aligns with evidence suggesting that adapting mHealth interventions to local technological preferences and capacities is essential for sustainability [51].

In this study, WhatsApp was not implemented as an isolated tool; rather, it was combined with other communication components such as the use of loudspeakers and community visits during the IC application period. This strategy aligns with the WHO Communication for Behavioural Impact framework, which emphasizes the combination of interpersonal communication and community mobilization, often supported by mass media, to facilitate the adoption and maintenance of preventive behaviours [52]. A recent systematic review highlighted that this approach could improve knowledge, attitudes, and practices, leading to reductions in mosquito breeding sites and, subsequently, dengue incidence [53]. In this study, households exposed to multiple communication components, including WhatsApp, experienced greater improvements in preventive practices and larger reductions in vector densities compared to those with little exposure. Although these effects cannot be fully attributed to WhatsApp alone, its integration into vector control programmes may support dengue, chikungunya, and Zika prevention.

When interpreting these behavioural changes, it is important to consider the possible role of sociodemographic factors and previous household habits. In Colombia, educational level may be particularly relevant, as it can influence the understanding and adoption of preventive recommendations [54]. In our study, however, education and other sociodemographic factors were included as covariates in the adjusted models and were not significantly associated with most preventive practices. Households that were already adopting preventive measures before the intervention were more likely to maintain these behaviours after the intervention. Importantly, not all intervention-related behaviours reflected pre-existing household habits. IC application in laundry tanks represented a new practice for the community, requiring households to prepare the tank and allow the product to be applied. This component was assessed through direct field observation, providing additional evidence of active household participation beyond self-reported practices.

This active participation was one of the main indicators of community engagement observed in the study, particularly among households exposed to WhatsApp messaging. Previous research has shown that community engagement in mHealth interventions can be strengthened through the use of reminders, personalized messages, timely feedback, opportunities for peer support within the app, and incentives or other forms of compensation [55, 56]. In our study, several of these features were incorporated into the WhatsApp group intervention. Although no monetary incentives were provided to the community, the provision of IC at no cost may have been perceived as a non-financial benefit. As IC was provided in both intervention groups (CV + WA group and CV-only group), the higher engagement observed in the CV + WA group may be partly attributed to the additional elements incorporated into the WhatsApp intervention.

Technical issues remain a challenge in digital health interventions. Limited infrastructure, low network access, unstable internet connectivity, and incompatibility between apps and devices have been identified as common barriers to the implementation of app-based interventions [57–59], particularly in low- and middle-income countries [60]. Although some of these barriers cannot be fully eliminated, they may be mitigated in settings where WhatsApp is widely used and where mobile operators offer low-cost bundles or zero-rated access for specific applications. In Colombia, for example, many operators include zero-rated access to WhatsApp and Facebook in their postpaid and prepaid mobile plans [61], which may support continued use and lower cost-related barriers.

Data protection is another critical issue in digital interventions, particularly given the variation in ethical standards and regulatory frameworks across countries [62, 63]. Although WhatsApp is a familiar app to many users, its use in health-related programmes raises challenges related to privacy, informed consent, and record keeping [64]. WhatsApp may not fully meet the requirements of health data protection frameworks [65]. Legal requirements for data storage and protection are complex and depend on the legislation of each country [64]. For the implementation of WhatsApp, this study followed the national data protection law, which regulates the processing of personal data and requires informed consent and appropriate security safeguards [66]. However, future work should be accompanied by clear protocols and guidelines for data management and protection.

Despite these challenges, this study provides pioneering evidence that incorporating WhatsApp into a community-based public health strategy can support the uptake of household-level vector control interventions in endemic urban settings. This strategy may complement routine vector control activities by improving preventive practices, supporting vector control operations, and enhancing community engagement. This study, together with another recent study, supports the idea that WhatsApp is a feasible tool for arboviral disease prevention [51]. However, important questions remain regarding its scalability and sustainability within routine public health programmes. Therefore, future research should include economic and operational analyses to assess the long-term sustainability and scalability of adding WhatsApp to vector control programmes for arboviral disease prevention.

### Limitations

Although this study adds valuable evidence on the incorporation of WhatsApp as a digital tool for dengue, chikungunya, and Zika prevention, some contextual and methodological considerations should be taken into account when interpreting the findings. First, the intervention was mainly designed to promote household preventive behaviours, with a strong emphasis on the promotion of IC application in tanks. Therefore, generalizability to other prevention practices may be limited, particularly for behaviours that are promoted in isolation or require different enabling conditions.

Second, this was an implementation-oriented, exploratory study using a quasi-experimental design. A randomized controlled trial was not feasible given the community-based and behavioural nature of the intervention and operational constraints. Although multivariable models were used to adjust for potential confounding factors, residual confounding cannot be completely excluded. The findings should be interpreted within the context of a pragmatic evaluation conducted under real-world programme conditions.

Third, preventive practices were evaluated using self-reported data and may be subject to recall and social desirability bias. Moreover, participant identity and message exposure could not be fully verified throughout the intervention due to logistical constraints and data protection considerations. However, the inclusion of entomological indicators and direct observation related to IC application provided objective measures that strengthened the assessment beyond self-report alone.

Finally, the intervention was conducted in urban communities in Colombia with high smartphone penetration and a generally favourable attitude toward receiving preventive messages. As participation required access to a smartphone, use of WhatsApp, and the ability to receive and read messages, selection bias cannot be ruled out. Transferability to settings with lower digital access, lower digital literacy, different communication preferences, or weaker connectivity may therefore be limited and should be assessed in future studies.

## Conclusions

The integration of WhatsApp into a community-based arboviral disease prevention strategy demonstrated the potential of digital tools to strengthen community engagement in health programmes. Improvements in preventive practices and reductions in vector abundance were observed when WhatsApp was combined with community visits. These findings suggest that adding WhatsApp messaging to vector control programmes may support preventive actions, particularly those requiring household preparation and active participation. WhatsApp was also found to be acceptable an tool for community-based health interventions. However, further research is needed to assess implementation processes and the scalability of the intervention.

## Supplementary Information

Supplementary Material 1.

Supplementary Material 2.

Supplementary Material 3.

Supplementary Material 4.

Supplementary Material 5.

## Data Availability

The datasets generated and/or analysed during the current study are available in the bwSync&Share repository, https://bwsyncandshare.kit.edu/s/G8Fi63cZLoRESL8 .

## Abbreviations

BI: Breteau Index
CI: Container Index
CV: Community visits
CV only: Community visits-only group
CV + WhatsApp: Community visits plus WhatsApp group
DANE: National Administrative Department of Statistics
IDS: Departmental Health Institute of Norte de Santander
DiD: Difference-in-Differences
HI: House Index
IC: Insecticidal coating
Minciencias: Ministry of Science, Technology and Innovation
MoH: Ministry of Health
PPC: Percentage-point change
PPI: Pupae per Person Index
PI: Principal investigator
WA: WhatsApp
WHO: World Health Organization
