# Supplementary material for: Using a digital health tool to strengthen community engagement in arboviral disease prevention: an implementation study in Colombia
Source: BMC Public Health. 2026 Aug 4;26:2261. doi: 10.1186/s12889-026-28576-7 (PMC13435411; doi:10.1186/s12889-026-28576-7)
Supplement: Supplementary file 1 [file 12889_2026_28576_MOESM1_ESM.docx]

**Description of WhatsApp messages used during the application of the insecticidal coating**

| # of message | WhatsApp Broadcast Messages | Message category | Purpose |
| --- | --- | --- | --- |
| 1 | 📱 Welcome to the Digital Health Project, supported by the **[name of the health authority].** Over the coming weeks, you will receive preventative messages about dengue, chikungunya, and zika prevention and the **protective coating** for laundry tanks. Our team will visit your **[neighbourhood name]** on **[date]** to provide the intervention. Thank you for participating in this project. | Health communication  Welcome message | Introduce participants to the project and explain upcoming activities |
| 2 | 👩 **Meet [name of community woman]**, a participant from **[neighbourhood name]**. She has already painted her laundry tank with the protective coating. Like [name of woman], you can also help prevent dengue. On **[date]**, the health team will visit your neighbourhood to support the application | Health promotion  Storytelling / social modelling | Motivate participation through a community example |
| 3 | 📢 **Attention community members!** Please empty your laundry tank as much as possible before the health team visit. The protective coating is applied only to the internal walls of the laundry tank. **[Date of the application in each neighbourhood]** | Health promotion  Preparation before IC Application | Remind households to prepare the laundry tank |
| 4 | Have you already emptied your laundry tank❓If not, please try to empty it as much as possible so that the internal walls can be painted. **Tomorrow**, the health team will visit your home to provide the **protective coating** and support its application. Remember: this applies only to laundry tanks. | Behaviour modification  Reminder before IC Application | Remind households to empty the laundry tank before the health team visit |
| 5 | 🚨 **Today is the day!** The health team will visit your home to support the application of the **protective coating**. Please allow access to the team and participate in this dengue prevention activity. **[Date of the application in each neighbourhood]** | Behaviour modification  Same-day reminder | Encourage household participation on the application day |
| 6 | ✅ After painting your laundry tank, please let it dry completely for one day. The next day, you can use it normally. Thank you for helping prevent dengue. | Behaviour modification  Post-application reminder | Reinforce correct drying after application |
| 7 | 👏 **Thank you to families from [neighbourhood name]**.**!** Your support in applying the **protective coating** contributes to dengue prevention and community wellbeing. | Health communication  Community appreciation | Thank households for participation |
| 8 | Thank you for informing us. We are sorry that your household did not receive the **protective coating** or application support. Please send us your address so we can verify the information and arrange a follow-up visit. | Health communication  Follow-up for missed households | Respond to households that reported no visit or no coating |
| 9 | **🌞Good morning!** We would like to hear your opinions about the **protective coating**. Have you noticed any differences since it was applied?  📸 Feel free to send us a photo of your tank. Your opinions are very valuable to us and will help improve future dengue prevention activities.  **🤝Together against dengue, chikungunya, and zika** 🚫🦟 | Behaviour modification  Feedback and user experience collection | Gather participant opinions, experiences, and perceptions after IC application |
| 10 | The laundry tank is often one of the main **mosquito breeding sites** in your community. If you allowed the protective coating to be applied, thank you for your commitment and participation in dengue prevention activities. 🤝**Together against dengue, chikungunya and zika** 🚫🦟 | Health education  Community reinforcement | Reinforce the link between laundry tanks and mosquito breeding |

Note: Insecticidal coating was referred to as “protective coating” to make the message more understandable for participants.
