# Supplementary material for: Using a digital health tool to strengthen community engagement in arboviral disease prevention: an implementation study in Colombia
Source: BMC Public Health. 2026 Aug 4;26:2261. doi: 10.1186/s12889-026-28576-7 (PMC13435411; doi:10.1186/s12889-026-28576-7)
Supplement: Supplementary file 2 [file 12889_2026_28576_MOESM2_ESM.docx]

**Household questionnaire**

With your permission, I would like to ask a few brief questions that will only take a short amount of your time. If at any point you feel uncomfortable, you may choose not to answer or stop the interview whenever you wish.

Group: _____________ Household code: ________________	Interviewer code: ___

**Part A: Sociodemographic information**

1. **¿Could you please provide the following information about all household members, including yourself, in the table below?**

| Member | Age | Sex  1=F  2=M  3=O | Education level  Primary=1  secondary=2 University=3 No= 4 | In the last 12 months, did you or anyone in your household have arbovirus?  1=Dengue 2=Zika 3=Chikunguna 4=No | Which month? | Did you visit the doctor or health center?  1=Yes 2=No | Where do you stay most of the time (7 am to 5 pm)?  1=Inside your area, 2=Outside your area |
| --- | --- | --- | --- | --- | --- | --- | --- |
| **surveyed** |  |  |  |  |  |  |  |
| 2 |  |  |  |  |  |  |  |
| 3 |  |  |  |  |  |  |  |
| 4 |  |  |  |  |  |  |  |
| 5 |  |  |  |  |  |  |  |
| 6 |  |  |  |  |  |  |  |
| 7 |  |  |  |  |  |  |  |
| 8 |  |  |  |  |  |  |  |
| 9 |  |  |  |  |  |  |  |
| 10 |  |  |  |  |  |  |  |
| 11 |  |  |  |  |  |  |  |
| 12 |  |  |  |  |  |  |  |

Part B: Preventative practices and communication preferences

2. **Do you use any type of vector control method to prevent dengue, chikungunya or Zika?** *(Multiple answers allowed)*

1= Fish in tanks	2 = Chlorine in tanks	3=Covering tanks	4=Cleaning tank (one per week)

5=Management of small breeding sites 6=other:_ 7= No method	8=I do not know

3. **Has health workers applied any type of insecticide powder or tablet to your tank this year?**

1= Yes	2= No	3= I do not know

4. **Which water storage containers do you use for water consumption?**

1= Ground tank	2=Tap water	3=Other containers, specify____________	4=None

5. **Do you know where mosquitoes lay their eggs or breed?**

1=Clean	2=Dirty water	3=Grass or pasture	4=I do not know

6. **Through which of the following media would you like to receive information about dengue?** *(Multiple answers allowed)*

1=TV 2=Radio 3=Mobile phone 4=internet (computer) 5=Newspaper 6=Flyers 6=Home visits by a health worker 7=Loudspeakers 8=I don’t know

7. **Would you be willing to apply a transparent coating free of charge as a control method in your tank?**

1 = Yes	2=No	3= I do not know

**Part C: Community willingness to receive the insecticidal coating**

Part D: Post intervention (intervention groups only)

8. **Was your water tank painted with the insecticidal coating?**

1=Yes	2=No	3=I do not know

9. **Have you noticed any side effects?**

1= Damage	2=it does not look good	3=Bad smell	4=More larvae/puape	5=health problems 6=None

10. **Would you recommend the insecticidal coating to other people?**

1= Yes	2=No	3= I do not know

11. **Have you noticed a reduction in mosquitoes?**

1=Yes	2=No	3= I don’t know

Part E: Post-intervention (WhatsApp group only)

12. **Did you or any member of your family receive WhatsApp messages?**

1=Yes	2=No	3=I do not know

13. **How would you rate the WhatsApp messages on a scale from 1 to 5?**

*(1 = strongly disagree, 2 = disagree, 3 = neutral, 4 = agree, 5 = strongly agree,)*

| **Items** | **1** | **2** | **3** | **4** | **5** |
| --- | --- | --- | --- | --- | --- |
| a. I think that receiving the messages is enjoyable |  |  |  |  |  |
| b. I think that messages are a good source of information for promoting healthy behaviours |  |  |  |  |  |
| c. I think that messages provide me with the information I need for vector control. |  |  |  |  |  |
| d. I think that messages are annoying. |  |  |  |  |  |
| e. I am satisfied with the WhatsApp messages |  |  |  |  |  |

14. **Would you recommend WhatsApp messages to your friends or family?**

1=Yes	2=No	3= I do not know

**Thank you for your support**
