# Supplementary material for: Using a digital health tool to strengthen community engagement in arboviral disease prevention: an implementation study in Colombia
Source: BMC Public Health. 2026 Aug 4;26:2261. doi: 10.1186/s12889-026-28576-7 (PMC13435411; doi:10.1186/s12889-026-28576-7)
Supplement: Supplementary file 3 [file 12889_2026_28576_MOESM3_ESM.docx]

**Larval/pupal household survey**

**House code: ________	Group: _____	Health worker: _______________	Date: ___/____/_____**

**Building type:** House: _	Shop/store: _ Health unit: _ Church: _	School: _	Restaurant: _ other**:** _____________	Public space:__

| **#** | | ***Large water tanks** | | | | | | **Small water containers** | | | | | | | | | | ***** **Details of each tank** | | | | | **Larva and pupae** | | ***Measures** | | | |
| --- | --- | --- | --- | --- | --- | --- | --- | --- | --- | --- | --- | --- | --- | --- | --- | --- | --- | --- | --- | --- | --- | --- | --- | --- | --- | --- | --- | --- |
|  | | *Ground tank | | | | Storage containers | Elevated tanks | Buckets | Cans / small containers / Jars | Flowerpots | Tires | Sumps / sewers | Bottles (with water) | Gasoline bottles (*Pimpinas*) | Natural breeding site | Other: | Other: | Volume capacity (L) | Filling level (1=Empty, 2=partially filled, 3=Full) | Tank location  (1=Inside, 0=Outside) | Shading  (1=Yes, 2=partial, 3=No) | Covered tank  (1=Yes, 2=partial, 3=No) | Larvae | Pupae | Length (cm) | Width (cm) | Height (cm) |  |
|  |  | Concrete | Plastic | Ceramic | Other |  |  |  |  |  |  |  |  |  |  |  |  |  |  |  |  |  | Positive (+),  Negative (-) | Pupae count |  |  |  |  |
| **Each water tank has its row (Water tank such as bottles etc. can be put together)** | 1 |  |  |  |  |  |  |  |  |  |  |  |  |  |  |  |  |  |  |  |  |  |  |  |  |  |  |  |
|  | 2 |  |  |  |  |  |  |  |  |  |  |  |  |  |  |  |  |  |  |  |  |  |  |  |  |  |  |  |
|  | 3 |  |  |  |  |  |  |  |  |  |  |  |  |  |  |  |  |  |  |  |  |  |  |  |  |  |  |  |
|  | 4 |  |  |  |  |  |  |  |  |  |  |  |  |  |  |  |  |  |  |  |  |  |  |  |  |  |  |  |
|  | 5 |  |  |  |  |  |  |  |  |  |  |  |  |  |  |  |  |  |  |  |  |  |  |  |  |  |  |  |
|  | 6 |  |  |  |  |  |  |  |  |  |  |  |  |  |  |  |  |  |  |  |  |  |  |  |  |  |  |  |
|  | 7 |  |  |  |  |  |  |  |  |  |  |  |  |  |  |  |  |  |  |  |  |  |  |  |  |  |  |  |
|  | 8 |  |  |  |  |  |  |  |  |  |  |  |  |  |  |  |  |  |  |  |  |  |  |  |  |  |  |  |
|  | 9 |  |  |  |  |  |  |  |  |  |  |  |  |  |  |  |  |  |  |  |  |  |  |  |  |  |  |  |
|  | 10 |  |  |  |  |  |  |  |  |  |  |  |  |  |  |  |  |  |  |  |  |  |  |  |  |  |  |  |
|  | 11 |  |  |  |  |  |  |  |  |  |  |  |  |  |  |  |  |  |  |  |  |  |  |  |  |  |  |  |
|  | 12 |  |  |  |  |  |  |  |  |  |  |  |  |  |  |  |  |  |  |  |  |  |  |  |  |  |  |  |
|  | 13 |  |  |  |  |  |  |  |  |  |  |  |  |  |  |  |  |  |  |  |  |  |  |  |  |  |  |  |
|  | 15 |  |  |  |  |  |  |  |  |  |  |  |  |  |  |  |  |  |  |  |  |  |  |  |  |  |  |  |
|  | 16 |  |  |  |  |  |  |  |  |  |  |  |  |  |  |  |  |  |  |  |  |  |  |  |  |  |  |  |
|  | 17 |  |  |  |  |  |  |  |  |  |  |  |  |  |  |  |  |  |  |  |  |  |  |  |  |  |  |  |
|  | 18 |  |  |  |  |  |  |  |  |  |  |  |  |  |  |  |  |  |  |  |  |  |  |  |  |  |  |  |

- This information is exclusively for laundry tank

Comment: ___________________________________________________________________________________________________________________
