# Supplementary material for: Using a digital health tool to strengthen community engagement in arboviral disease prevention: an implementation study in Colombia
Source: BMC Public Health. 2026 Aug 4;26:2261. doi: 10.1186/s12889-026-28576-7 (PMC13435411; doi:10.1186/s12889-026-28576-7)
Supplement: Supplementary file 4 [file 12889_2026_28576_MOESM4_ESM.docx]

Field form for the coating application

**Investigator code:**	**Date**: __/__/____

**Health worker/interviewer code:** __________	**Municipality:** _________

*Instructions: Inform the participant about the WhatsApp strategy supported by the Departmental Ministry of Health of Norte de Santander. Explain that participation is voluntary and that, if they agree, they will be asked to register their WhatsApp contact to receive occasional preventive and promotional messages on working days for up to 9 months. Also explain that their personal data will be kept confidential, will not be shared without permission, and that they may withdraw at any time without any consequences.*

| **code** | **Area** | **Sex (Female, male, other)** | **Code Area** | **WhatsApp contact (Yes/No)** | **Paint tank (Yes/No)** | **Type Tank (Cement, Plastic, Bricks, ceramic,others)** | **Clean and empty the tank (Yes/No)** | **Comment** |
| --- | --- | --- | --- | --- | --- | --- | --- | --- |
| **1** |  |  |  |  |  |  |  |  |
| **2** |  |  |  |  |  |  |  |  |
| **4** |  |  |  |  |  |  |  |  |
| **4** |  |  |  |  |  |  |  |  |
| **5** |  |  |  |  |  |  |  |  |
| **6** |  |  |  |  |  |  |  |  |
| **7** |  |  |  |  |  |  |  |  |
| **8** |  |  |  |  |  |  |  |  |
| **9** |  |  |  |  |  |  |  |  |
| **10** |  |  |  |  |  |  |  |  |
| **11** |  |  |  |  |  |  |  |  |
| **12** |  |  |  |  |  |  |  |  |
| **13** |  |  |  |  |  |  |  |  |
| **14** |  |  |  |  |  |  |  |  |
| **15** |  |  |  |  |  |  |  |  |
| **16** |  |  |  |  |  |  |  |  |
| **17** |  |  |  |  |  |  |  |  |

***Thank you for your participation in this study***
